# Liver Resection as Part of Cytoreductive Surgery for Ovarian Cancer

**DOI:** 10.1089/gyn.2019.0074

**Published:** 2020-03-31

**Authors:** Jorge Luna-Abanto, Luis García Ruiz, Jheff Laura Martinez, Manuel Álvarez Larraondo, Vladimir Villoslada Terrones

**Affiliations:** ^1^ Department of Surgical Oncology, Instituto Nacional de Enfermedades Neoplásicas, Lima, Perú.; ^2^ Department of Gynecology Oncology, Instituto Nacional de Enfermedades Neoplásicas, Lima, Perú.; ^3^ School of Human Medicine, National University of Cajamarca, Cajamarca, Perú.

**Keywords:** cytoreductive surgery, ovarian cancer, metastasis, liver

## Abstract

***Objective:*** The aim of this study was to describe and evaluate the safety of hepatic resections for ovarian cancer liver metastases and the benefit in terms of survival as part of cytoreductive surgery among peritoneal seeding and parenchymal metastases.

***Materials and Methods:*** Data were reviewed retrospectively from patients who underwent liver resection as part of cytoreductive surgery for ovarian cancer at the Instituto Nacional de Enfermedades Neoplásicas, in Lima, Perú, from January 2009 to December 2017.

***Results:*** From January 2009 to December 2017, 1211 patients underwent surgical cytoreduction for ovarian cancer; 39 of these patients had liver resection as part of their surgical treatment, with 9, 17, and 13 patients receiving primary, secondary, and tertiary, resections, respectively. The mean age of the patients was 46, the majority (87%) had stage III/IV ovarian cancer. In addition, 21 patients had parenchymal metastasis resections, and 95% of the patients had Dindo–Clavien I and II grade complications. The 30-day mortality rate was 0.

***Conclusions:*** Liver resection for advanced ovarian cancer is a safe procedure for primary up to quaternary cytoreduction and may confer survival benefits to patients.

## Introduction

Ovarian cancer is the leading cause of death from gynecologic malignancies worldwide, with an estimated incidence of 22 000 new cases and 14 000 deaths in the United States during 2013.^1^ In Perú, according to the Metropolitan Lima cancer registry 2004–2005, ovarian cancer is the eighth most-frequent neoplasm and the second most-frequent gynecologic neoplasm after cervical cancer, representing the second most-frequent cause of death.^2^ Most patients are diagnosed at advanced stages, as no effective screening tests exist and symptoms are discreet.^1,2^ Ovarian cancer can spread through the intraperitoneal, lymphatic, and hematogenous routes. The most common sites of metastatic disease are the peritoneum, liver, and lymph nodes. It is known that patients with advanced ovarian cancer, regardless of the site of metastasis have poor prognoses.^3^ Winter et al. reported that hepatic parenchymal metastases represent 18% of these cancers and were the second most-frequent cause of stage IV disease.^4^ In addition, another study showed that liver metastases were found in up to 50% of patients who died from ovarian cancer.^1,5^ Therefore, liver metastases are common findings in these patients.

Cytoreduction, followed by platinum-based chemotherapy, is the current standard treatment for ovarian cancer.^3,5^ A systematic meta-analysis showed that with every 10% increase in optimal cytoreduction, there is a 5.5% increase in survival.^5^ In this context, some studies have explored the implementation of upper abdominal surgery to achieve complete macroscopic debulking. The surgical technique and postoperative care have improved, achieving safer and more extensive surgeries (multivisceral resections). However, management of liver metastases still represents a limit for complete surgical treatment. This is evidenced in the few studies evaluating the safety and benefit of hepatic resections in patients with advanced ovarian cancer.^1,5,6^

## Materials and Methods

Data were retrospectively reviewed from patients who underwent surgical cytoreduction for ovarian cancer that included liver resection at the Instituto Nacional de Enfermedades Neoplásicas (Lima, Perú) from January 2009 to December 2017. Patients with primary, secondary, and tertiary or more cytoreductions were included in the analysis. The collected information included the patient's age at diagnosis, International Federation of Gynecology and Obstetrics (FIGO) staging; primary tumor histology and grade (well [G1], moderately [G2], or poorly [G3] differentiated carcinomas); neoadjuvant chemotherapy; associated organ resections during cytoreductive surgery; and the number, maximum dimensions, type of liver metastasis, residual disease (R), and margin status. Liver resection security was evaluated by complication grades (according to the Clavien–Dindo system^7^) length of hospitalization, and 30-day mortality.

Disease-free survival and overall survival were calculated from the date of surgery; death status was obtained from the Instituto Nacional de Estadística e Informática registry. The differences between the different subgroups were analyzed with nonparametric tests (Fisher, Wilcoxon). The differences between groups in terms of disease-free survival and overall survival were analyzed by a log-rank test and were considered significant if *p* < 0.05. Kaplan–Meyer survival curves were used. Statistics and graphics were made, using SPSS software, version 22.0. This research had approval from the Instituto Nacional de Enfermedades Neoplásicas ethics committee (INEN 19-04).

## Results

From January 2009 to December 2017, 1211 patients underwent surgical cytoreduction for ovarian cancer, of whom 39 patients had liver resection as part of their surgical treatment, and 21 patients had parenchymal metastasis resection. The mean age of these patients was 46, the majority (87%) had stage III/IV ovarian cancer. Of the 39 patients who had patients had liver resection as part of their cytoreductive surgeries, 9, 17, and 13 primary, secondary, and tertiary cytoreductions, respectively. In addition, 58% of the patients had epithelial-type ovarian carcinomas. Among the patients who did not undergo primary cytoreductions, 14 and 2 were platinum-sensitive and resistant, respectively. Other results included (Table 1):

**Table 1.** Characteristics of Patients Undergoing Liver Resection as Part of Cytoreductive Surgery

Liver metastasis type	Parenchymal	Peritoneal seeding
Mean age	49 (15–71)	43 (16–71)
FIGO stage		
I–II	3	2
III–IV	18	16
Neoadjuvant chemotherapy	18	13
Cytoreductive surgery		
Primary	3	6
Secondary	10	7
Tertiary and quaternary	8	5
Histologic type		
Epithelial	13	10
Germ	2	1
Stromal	6	6
Other types	0	1
Differentiation grade		
G1	2	1
G2	2	2
G3	6	4
Not reported	11	11

FIGO, International Federation of Gynecology and Obstetrics.

- 30% of patients had stromal tumors.
- 35 patients had single liver metastasis.
- Mean diameters were 4.38 cm for parenchymal metastasis and 4.55 cm for peritoneal seeding.
- R0 resection was accomplished in 61% of the patients.
- 33, 3, and 2 patients underwent minor hepatectomy, segmentectomy. and major hepatectomy respectively.
- Margin status was not reported in 76% of the cases.
- The most-frequent associated organ resection was the spleen, followed by the omentum and peritoneum.
- Mean length of hospital stay was 5 days (range 1–11 days).
- 26 and 13 patients had Clavien–Dindo scores of I and II, respectively.
- The 30-day mortality rate following cytoreductive surgery was 0 (Table 2).

**Table 2.** Intraoperative Findings, Types of Resection, and Early Postoperative Outcomes of Patients Undergoing Liver Resection as Part of Cytoreductive Surgery

Liver metastasis type	Parenchymal	Peritoneal seeding
Number of liver metastasis		
Single	18	17
Multiple	3	1
Diameter of liver metastasis	4.38 cm (1–11 cm)	4.55 cm (1–18 cm)
Type of resection		
R0	12	12
R1	6	5
R2	3	1
Type of liver resection		
Minor hepatectomy	16	17
Segmentectomy	3	0
Major hepatectomy	2	0
Margin status		
Free	3	1
Compromised	5	0
Not reported	13	17
Associated visceral resections		
Solid	5	2
Hollow	5	4
Peritoneum/omentum	4	8
Length of hospital stay	5.5 (3–9)	5 (1–11)
Morbidity (Clavien–Dindo)		
I	14	12
II	7	6

**FIG. 1.**
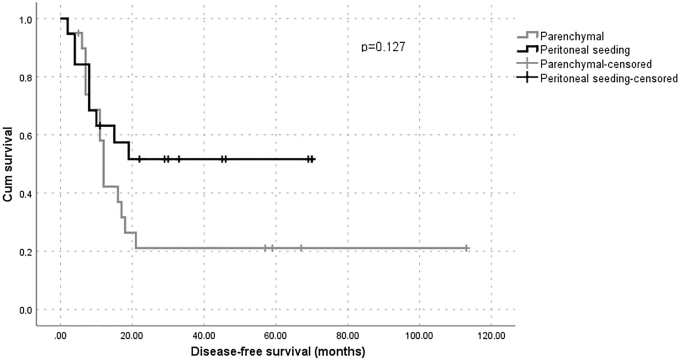
Disease-free survival of patients following liver resection for parenchymal and peritoneal seeding metastases from ovarian carcinoma. Cum, cumulative.

**FIG. 2.**
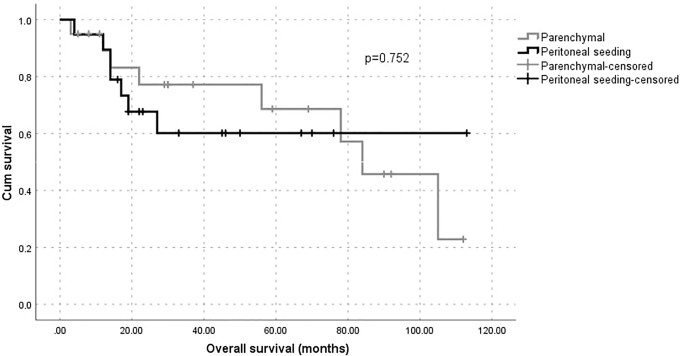
Overall survival of patients following liver resection for parenchymal and peritoneal seeding metastases from ovarian carcinoma. Cum, cumulative.

Disease-free survival was better in patients with peritoneal seeding metastasis; however this result was nonsignificant. The overall survival analysis showed no difference between patients who underwent to cytoreductive surgery—including parenchymal and peritoneal seeding and liver-metastasis resections (Figs. 1 & 2). All patients had at least 12 months of follow-ups.

## Discussion

Patients diagnosed with ovarian cancer present frequently in advanced stages, usually with proven metastases.^8^ Once patients are diagnosed and staged, the next step is cytoreductive surgery, which will normally include hysterectomy, bilateral salpingo-oophorectomy, omentectomy, and resection of all metastatic lesions. Approximately 40% of patients with advanced ovarian cancer have bulky tumors in the upper abdomen, including the diaphragm, stomach, and liver.^9^

The effect of this procedure is the benefit of all macroscopic tumor removals, and depends on the characteristics of the tumors, the sizes of the lesions, the number of metastasis, and the viability of multivisceral resection,^10^ A strong relationship between survival and R0 cytoreduction has been proven; this benefit extends to secondary, tertiary and quaternary cytoreductions.^1^ The management of liver metastases still represents a limit for a complete surgical treatment; few reports agree that liver resection as part of cytoreductive surgery for ovarian cancer is safe and may offer survival benefits.^1–3,5,6,8^ However, a 2015 report stated that liver resection in these patients was associated with life-threatening complications and impaired liver function,^11^ and some researchers have questioned the benefits of surgical management of non-colorectal, nonneuroendocrine liver metastasis.^5,8^

The average ages of the patients with parenchymal and peritoneal seeding metastasis were 47 and 45 years, respectively. In 2005, Loizzi et al. reported on 29 patients, with a mean age of 59, with primary and recurrent epithelial ovarian cancer with hepatic involvement.^12^ Similar data were published by Bacalbasa et al. and Kolev et al. who reported mean ages at the time of liver resection of 53 and 62, respectively.^1,7^ It is evident that the majority of patients studied was between ages 40 and 60, probably because this group represent a better patient for this kind of procedure. The majority of the current study's patients had epithelial ovarian cancer (58%), followed by stromal (30%) and germ-cell tumors (7%). Of these current patients 88% of were diagnosed as having stages III and IV ovarian cancer. With similar results, Bacalbasa et al. reported that the majority of patients who underwent hepatic resection had advanced ovarian cancers.^1,5^

Of the patients in the current series, 80% had received previous chemotherapy; however the effect of this in the surgical outcomes has not been established.^5,11^ Kolev et al. reported that all patients undergoing liver resection during secondary cytoreduction due to epithelial ovarian cancer were able to receive chemotherapy; this means that the magnitude of liver resection did not impair the liver function enabling the patients to receive systemic treatment.^7^ Therefore, the benefits of neoadjuvant chemotherapy, followed by interval cytoreduction that included liver resection is an alternative for patients who are not able to undergo primary cytoreduction.^5^ In addition, the current study showed that the majority of patients had secondary cytoreductions (17 patients), followed by tertiary and primary cytoreductions; these patients were able to continue with systemic therapy independently of the magnitude of their liver resections. Some researchers recommend considering liver resections during secondary cytoreduction, given that this seems to improve survival, is being feasible, and is safe.^6^ In contrast, other studies showed this benefit in primary, tertiary, and even quaternary cytoreductions.^1,5,11^

The state of sensitivity to platinum as well as the platinum-free intervals has not been studied in previous reports on the role of liver surgery as part of secondary cytoreductions.^1–3,5,6,8^. Platinum-sensitivity state seems to be an important variable when selecting the right patients for surgery. In the current series, the majority of patients with serous ovarian cancer (88%) who received chemotherapy prior surgery were classified as platinum-sensitive at the time of liver surgery, probably due to the selection of patients with better prognoses.

In the current series, 89% of the patients who underwent surgical cytoreduction had single liver metastases. Merideth et al. reported 65% of patients with single liver metastasis resection.^10^ Niu et al. reported 60 patients of whom 60% had multiple liver metastases resection.^13^ In addition, Kolev et al. reported 56% of multiple liver resections with good postoperative results in a series of 29 patients.^6^ Single or multiple liver metastasis resection comprise a variable; this difference probably depends on the center's experience and the resectability of the liver metastases. The average sizes of the liver lesions were 4.38 cm and 4.55 cm for metastases of parenchymal and peritoneal origin, respectively. However, the range of values was variable between 1 and 18 cm in size. Research by several authors indicates that the average size of resected liver metastases varies between 4.5 cm and 5 cm.^5,10,14,15^ In addition, Sal et al. reported that 41% of the resections included liver metastases of >10 cm in diameter.^15^ No differences have been demonstrated between the size of liver lesions according to their origin (parenchymal or peritoneal).^1^ The current authors found no significant difference between the mean sizes of liver metastases.

Optimal cytoreduction was achieved in the majority of patients (89%). Merideth et al. reported an 80% rate of optimal cytoreduction, in their series; these researchers reviewed the records of 26 patients who underwent liver resections as part of cytoreductive surgeries.^10^ Kolev et al. analyzed the records of 27 patients who underwent secondary cytoreduction; 92% of patients had optimal surgery.^6^ Optimal cytoreduction is a well-known factor for survival; these results correlate with the selection of patients and could be considered as surgical quality control. Minor liver resections were the most-frequently performed operations in the current series; similar results were found by other researchers.^1,3,15^ In contrast, anatomical liver resections seem to have a small role in the management of advanced ovarian cancer; however, this is yet to be established. A free liver-resection margin does not seem to influence the outcome of these particular patients according to the concept of optimal cytoreduction for ovarian cancer.^16^

The safety of liver resection as part of cytoreductive surgery has been demonstrated in few studies that have reported 0 mortality within 30 days postoperatively.^1,10^ In agreement with them, the current study had 0 mortality at 30 days postoperatively; this demonstrates the safety of this procedure in the current authors' center. Clavien–Dindo complications, categories I and II, occurred in 24 and 13, respectively. There was a mean hospital stay of 5 days, which was similar to the data reported by Kolev et al.,^6^ and Neumann et al.^16^ Benedetti Panici et al. reported that the mean hospital stay of complicated patients was 20 days; in that series, 17.8% had grade I and II Clavien–Dindo scores.^11^ This finding is consistent with the current evidence and demonstrates the safety of the procedure performed by a qualified team.^1,17,18^ However, life-threatening complications associated with liver resection have been reported; among them, the most frequent was bleeding followed by liver failure.^19^

Studies from 2009 onward have demonstrated an improvement in disease-free survival and total survival of patients undergoing hepatic metastasectomy regardless of the types of primary neoplasias.^7,9,20–25^ Based on previous studies, the mean total survival for patients with stage IV ovarian epithelial cancer varied between 15 and 29 months, with a total survival of 20% at 5 years.^18^ In the current study, disease-free survival was better in patients with peritoneal seeding metastasis; however this result was nonsignificant and the overall survival analysis showed no difference among these patients. In contrast, Bacalbasa et al. reported a clear difference in disease free-survival and overall survival in favor of the peritoneal seeding liver metastasis group.^1^ Rodríguez et al. argued that this advantage in disease-free survival and overall survival, described in some reports,^1,7,20–24^ was due to an indirect effect, because these patients could have gained the benefit of complete cytoreduction that could have influenced the prognoses.^26^

## Conclusions

Consistent with previous reports, the current authors' institutional experience with liver resections during cytoreductions for advanced ovarian cancers was associated with acceptable perioperative morbidity and mortality, and could offer survival benefits if complete resections are achieved. In patients with liver metastases via hematogenous spread and peritoneal seeding, a better prognosis in favor of the latter category was found; however this was nonsignificant.
